# Severity of punctate white matter lesions in preterm infants: antecedents and cerebral palsy prediction

**DOI:** 10.1038/s41390-025-04157-z

**Published:** 2025-05-30

**Authors:** E. Melinda Mahabee-Gittens, Venkata Sita Priyanka Illapani, Beth M. Kline-Fath, Karen Harpster, Ashley Magnino, Stephanie L. Merhar, Nehal A. Parikh

**Affiliations:** 1 Division of Emergency Medicine, Cincinnati Children’s Hospital Medical Center, Cincinnati, OH USA; 2 University of Cincinnati College of Medicine, Cincinnati, OH USA; 3 Neurodevelopmental Disorders Prevention Center, Cincinnati Children’s Hospital Medical Center, Cincinnati, OH USA; 4 Perinatal Institute, Department of Pediatrics, Cincinnati Children’s Hospital Medical Center, Cincinnati, OH USA; 5 Department of Radiology, Cincinnati Children’s Hospital Medical Center, Cincinnati, OH USA; 6 Division of Occupational Therapy and Physical Therapy, Cincinnati Children’s Hospital Medical Center, Cincinnati, OH USA; 7 Department of Rehabilitation, Exercise, and Nutrition Sciences, College of Allied Health Sciences, University of Cincinnati, Cincinnati, OH USA; 8 Cincinnati Children’s Hospital Medical Center, Cincinnati, OH USA

## Abstract

**Background:**

The objectives were to investigate antecedent factors of punctate white matter lesions (PWML) severity on MRI at term-corrected age (CA) and to evaluate its ability to independently predict cerebral palsy (CP) in preterm infants.

**Methods:**

We studied infants born at ≤32 weeks’ gestational age [GA] with brain MRI at term CA, a standardized neuromotor exam to determine CP diagnosis, and composite scores from Bayley Scales of Infant and Toddler Development-III(BSID-III) at 2 years CA. MRIs with PWML were manually segmented and volume quantified with high reliability. We correlated >50 perinatal antecedent factors with PWML severity and conducted multivariable regression analyses to assess PWML ability to independently predict neurodevelopmental outcomes at age 2.

**Results:**

Of 392 infants, 28 (7.1%) had PWML; 339 (86%) were assessed at age 2, 39 (11.6%) had CP. Moderate-severe acute histologic chorioamnionitis (HCA), prenatal opioid use, and antenatal corticosteroids were independently associated with PWML severity. Increasing PWML severity was significantly predictive of CP (OR 2.12; 95% CI:1.34,3.37), independent of known predictors, but not BSID-III scores.

**Conclusions:**

Increasing burden of PWML was associated with two-fold risk of CP in preterm infants. We also identified HCA, prenatal opioids, and antenatal corticosteroids as modifiable risk and protective factors for PWML that may be amenable to prevention efforts.

**Impact:**

- Punctate white matter lesions (PWML) are commonly seen on MRI scans in preterm infants, yet the antecedent factors associated with PWML are not well characterized.
- While prior literature is conflicting on the ability of PWML to predict neurodevelopmental impairments, our study demonstrated that objectively quantified PWML are independently predictive of the development of cerebral palsy.
- We identified modifiable factors such as histologic chorioamnionitis as a risk factor and antenatal corticosteroids as a protective factor against PWML development.
- Our findings may facilitate earlier identification of infants at risk for PWML and cerebral palsy.

## Introduction

Preterm birth is a well-established risk factor for neurodevelopmental impairments (NDI).^1–4^ Cerebral palsy (CP) is a spectrum of life-long disorders of movement and posture with an overall prevalence of 2.8 per 1000 children in the United States and higher prevalence rates of 16.5 per 1000 children who were born with low birthweights of <2500 grams.^5^ In very preterm infants, approximately 10% develop CP and 32–42% develop minor motor abnormalities.^6,7^ Although these motor abnormalities are caused by abnormal brain development or injury during the fetal or neonatal period, children typically are not diagnosed with CP until 1–2 years of age or later.^8^ Cerebral white matter injury, which can be sensitively identified on magnetic resonance imaging (MRI) studies, commonly precedes the development of CP and other NDI.^9–11^

Punctate white matter lesions (PWML) are not readily visible on cranial ultrasound but are among the more commonly observed injuries that are present in 7–59% of preterm infants on early and/or later term-equivalent age conventional MRI scans.^10–16^ Prior research indicates that PWML may be associated with adverse neurodevelopmental outcomes, especially CP.^11,14,17–23^ However, these studies differed in their conclusion about the predictive relationships between PWML and CP. For example, Guo et al.^14^ found limited evidence of CP in preterm children who had PWML, but Jeon et al.^17^ found a positive association. These mixed findings may be due to heterogeneity in sample size, study design, cohort characteristics, PWML diagnosis definition, and/or outcome severity. Qualitative diagnosis of PWML exhibits moderate to poor reliability, thus resulting in measurement error. More objective assessment by measuring MRI lesion volume and the addition of known predictors of CP in prognostic models could definitively establish the ability of PWML to independently predict CP.

Objective diagnosis of PWML in infants could also facilitate accurate identification of risk and protective factors that antecede the development of PWML and proactive interventions that could improve outcomes. Yet, few studies have examined the perinatal factors that antecede the development of PWML.^15,22,24,25^ Prior studies have uncovered only a few antecedents and none of these have been externally validated. Moreover, modifiable risk factors such as antenatal opioid and tobacco use appear to be associated with PWML,^26,27^ but they have not been examined in large cohorts of preterm infants other than from this regional cohort (see below). Thus, our two primary study objectives were: (1) to investigate a comprehensive array of antenatal, perinatal, and postnatal risk and protective factors of objective quantitative diagnosis of PWML severity on MRI at term-equivalent age and (2) to evaluate the ability of this lesion to independently predict the development CP at 2 years corrected age (CA) in a well-characterized regional cohort of preterm infants.

### Study design and subjects

This is a secondary analysis of a prospective regional cohort called the Cincinnati Infant Neurodevelopment Early Prediction Study (CINEPS) that enrolled 395 preterm infants soon after birth between September 2016 and November 2019. All infants were enrolled from the following five level III/IV academic and community Greater Cincinnati-area neonatal intensive care units: Cincinnati Children’s Hospital Medical Center (CCHMC), University of Cincinnati Medical Center, Good Samaritan Hospital, St. Elizabeth’s Healthcare, and Kettering Medical Center. Infants were eligible if they were born at ≤32 weeks gestational age (GA) and excluded if they: had had known chromosomal or congenital anomalies that affected the central nervous system or cyanotic heart disease; remained hospitalized at 45 weeks postmenstrual age (PMA) so that all scans could be performed at CCHMC within our intended window of 39–44 weeks PMA. The CCHMC Institutional Review Board approved the study. The other hospitals’ Institutional Review Boards approved the study based on an established reliance agreement. Written informed consent was provided by caregivers of all infants.

### Brain MRI acquisition

All infants were scanned with a brain MRI between 39- and 44-weeks PMA with the same 3T MRI magnet (Philips Ingenia) and a 32-channel head coil. All infants were fed prior to the scan, MRI noise was minimized using neonatal silicone earplugs, and we used blanket swaddles and vacuum immobilization (MedVac; CFI Medical Solutions) to reduce motion/promote natural sleep for a successful scan. High-resolution T1-weighted images (TE/TR 3.4/7.3, flip angle 11°, resolution 1 × 1 × 1 mm^3^), axial T2 (TE/TR 66/18,567 ms, flip angle 90°, resolution 1 × 1 × 1 mm^3^), and susceptibility weighted images (SWI, TE/TR 7.2/29 ms, flip angle 17°, resolution 0.6 × 0.6 × 2 mm^3^) were obtained. An experienced neuroradiologist (BKF) read and scored all structural MRI injuries and maturational abnormalities per the scoring system developed by Kidokoro et al. and previously described.^28,29^ Because this global brain abnormality score (GBAS) included PWML, we created a modified score (GBASm) that excluded PWML.

### PWML volume

**Fig. 1:**
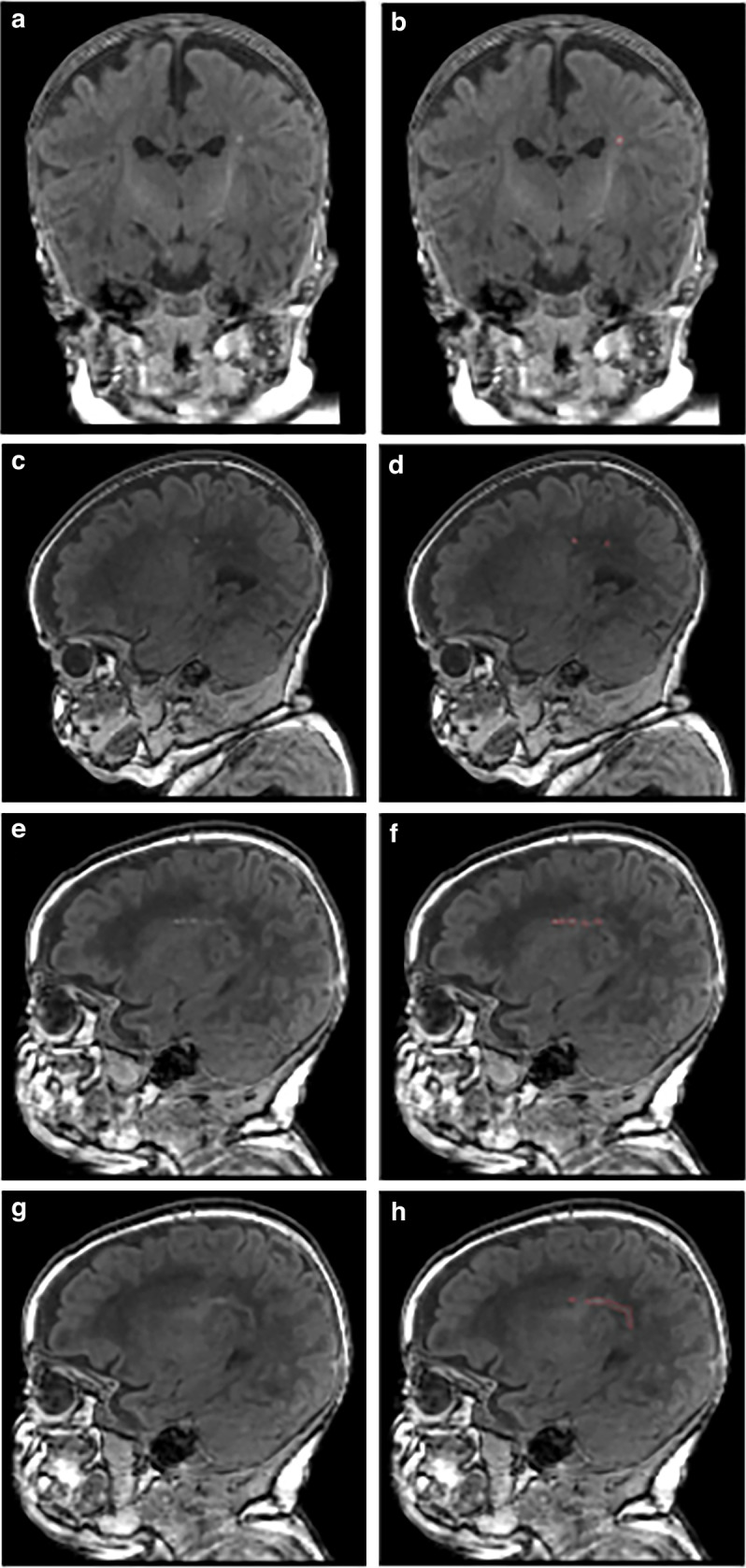
Punctate white matter lesions (PWML) with varying degrees of severity displayed using raw and segmented T1-weighted MRI scans (1 mm resolution) at term-equivalent age in three preterm infants. **a** 31 weeks’ gestational age (GA) infant with mild PWML (13 mm^3^; lowest tercile for volume [3–15 mm^3^]) in the left periventricular white matter. This infant also had another solitary lesion in the contralateral hemisphere (not shown); **b** corresponding segmented image; **c** 32 weeks’ GA infant with moderate PWML severity (71 mm^3^; middle tercile [23–71 mm^3^]) in the right central white matter. This infant also had a second contiguous sagittal slice with similar lesions and additional lesions in the contralateral hemisphere (not shown); **d** corresponding segmented image; **e** 28 weeks’ GA infant with severe PWML severity (185 mm^3^; highest tercile [75–338 mm^3^]) in the right central white matter; **f** corresponding segmented image; **g** same 28 weeks’ GA infant with more lesions in a contiguous sagittal slice; this infant also has a third contiguous slice with lesions and additional lesions in the contralateral hemisphere (not shown); **h** corresponding segmented image.

PWML was diagnosed qualitatively on anatomical MRI by a single experienced neuroradiologist as white matter non-hemorrhagic (SWI negative) T1 bright foci or white matter confluent non-hemorrhagic injuries (without T1 bright foci). T1-weighted images with qualitatively diagnosed PWML were manually segmented (Fig.1) as regions of interest (ROIs) by a trained rater as previously described.^30^ Briefly, using FSL (FMRIB), a single rater segmented voxels with bright signal intensity on T1-weighted images as PWML. All segmentations were reviewed for accuracy by an experienced neuroimaging researcher (NAP). Reliability testing was performed for both the radiologist (30 randomly selected MRI scans were reread four weeks later) and the trained rater (resegmented all PWML four weeks later while masked to prior results). MATLAB (R2019a) was used to calculate ROIs.

### Neurodevelopmental outcomes

All study children were invited back at 22–26 months CA for a standardized neuromotor exam, the Amiel-Tison Neurological Assessment, to determine a diagnosis of CP (none, mild, moderate, or severe), our primary outcome of interest.^31^ We used the Gross Motor Functional Classification System to further verify CP diagnosis and rate CP severity from level of I–V.^32^ A child with level I CP typically is able to walk 10 steps independently but with some gait abnormalities (including toe walking, asymmetric walking, or ataxic gait). A child with level V CP has physical impairments that limit voluntary control of movement and is wheelchair dependent. At this 2 years CA visit, we also tested all infants using the motor, cognitive, and language subscales of the Bayley Scales of Infant and Toddler Development, Third Edition (BSID-III) to derive a composite motor, cognitive, and language score (continuous secondary outcome).^33^ All BSID-III and Amiel-Tison examiners were trained and certified by the National Institutes of Child Health and Development Neonatal Research Network.^34^ The BSID-III composite sub-scores are standardized measures ranging from 40 to 160 and a mean (SD) of 100 (15). For children who were unable to be tested due to severe developmental delays, we assigned a cognitive score of 54, language score of 46, and/or a motor score of 46.

### Clinical antecedents

Information regarding maternal characteristics, antepartum, intrapartum and postpartum care were either extracted from hospital records using standardized definitions as previously described^35,36^ or through parental questionnaires completed during MRI visits.^27,30^ We defined social risk using a composite measure of six aspects of social status as per Roberts et al.^37^ with one key difference; we substituted occupation of primary provider, which we did not collect, with household income. The other five aspects of social status were family structure, primary caregivers’ education and employment, language spoken at home, and maternal age at birth. We also used the median score as the cut-point to define lower ($\le$3) vs. higher social risk (>3).^37^

### Statistical analysis

Baseline characteristics provided in Table 1 were compared between groups using Fisher’s exact test, Student’s t-test with unequal variances, or Wilcoxon rank sum test for categorical and continuous variables, as appropriate. We used multivariable logistic regression to correlate PWML severity with a diagnosis of CP at 2 years CA (primary outcome) while adjusting for five a priori selected known predictors of CP: GA, sex, moderate-severe bronchopulmonary dysplasia, GBASm, and PMA at MRI. In secondary analyses, we used multiple linear regression to evaluate the ability of PWML severity to predict BSID-III cognitive, language, and motor scores at 2 years CA, independent of known predictors. Since the BSID-III outcomes were continuous and the model had more degrees of freedom, in addition to the above five predictors, we also included antenatal steroids (ANS; full course), hypertensive disorders of pregnancy, moderate-severe acute chorioamnionitis (HCA), multiple births, postnatal sepsis (culture positive), and high-risk social status.^38,39^ We also tested the predictive value of PWML volume (mm3) by performing multivariable linear regression analysis while using the same 11 covariates as listed above. Because PWML volume was skewed, we used robust standard errors to meet the assumptions of linear regression. HCA was determined by a group of trained pathologists who used criteria by Redline et al.^40^ to clinically grade the placenta. Stage 2 and Stage 3 were classified as moderate and severe, respectfully. Placental pathology was missing for 41 (10%) subjects. We used nearest neighbor imputation algorithm to impute data for these missing subjects.^41^

**Table 1:** Baseline characteristics of mothers and their infants with and without punctate white matter lesions (PWML).

Baseline variables	Infants without PWML (*N* = 364)	Infants with PWML (*N* = 28)	*P*
Maternal smoking, *n* (%)	44 (12.1)	6 (21.4)	0.15
Maternal opioid use, *n* (%)	26 (7.1)	6 (21.4)	0.02
Maternal alcohol use, *n* (%)	29 (8.1)	0 (0)	0.24
Prenatal care, *n* (%)	333 (91.5)	24 (85.7)	0.30
Antenatal steroids, full course, *n* (%)	276 (75.8)	16 (57.1)	0.04
Magnesium sulfate, *n* (%)	308 (84.6)	22 (78.6)	0.42
Histologic chorioamnionitis, mod-severe, *n* (%)	50 (13.7)	7 (25.0)	0.10
Hypertensive disorders of pregnancy, *n* (%)	154 (42.3)	14 (50.0)	0.44
Multiple birth, *n* (%)	131 (35.9)	9 (32.1)	0.83
Gestational age, weeks, mean (SD)	29.2 (2.5)	29.9 (2.3)	0.15
Birth weight, grams, mean (SD)	1286 (452)	1400 (413)	0.18
Female sex, *n* (%)	170 (46.7)	13 (46.4)	1.00
Indomethacin prophylaxis, *n* (%)	15 (4.1%)	1 (3.6)	1.00
Clinical risk for babies II, median (IQR)	6.0 (7.0)	5.5 (4.5)	0.89
Sepsis, postnatal, *n* (%)	43 (11.8)	0 (0%)	0.06
Surgery requiring general anesthesia, *n* (%)	45 (12.4)	0 (0)	0.06
Retinopathy of prematurity, *n* (%)	21 (5.6)	1 (3.6)	1.00
BPD, mod-severe, *n* (%)	71 (19.5)	3 (10.7)	0.32
White matter injury, head ultrasound, *n* (%)	34 (9.3)	5 (17.9)	0.18
High-risk socioeconomic status, *n* (%)	164 (45.1)	10 (35.7)	0.43
PMA at MRI, weeks mean (SD)	42.8 (1.4)	42.1 (1.3)	0.02

To identify antecedent factors of PWML, we first performed bivariable ordered logistic regression between approximately 50 antenatal, intrapartum, and postnatal clinical factors with PWML severity as previously described.^36^ Briefly, variables that correlated with PWML (*P* < 0.15) were entered into a multivariable ordered logistic regression model in a manual backward stepwise manner, to evaluate their independent association with PWML severity. In addition, we used biological plausibility and knowledge of prior literature to guide variable selection. We carried out the analyses in a temporal fashion—antepartum first, intrapartum next, and postnatal last—so that postnatal covariates would not displace variables from earlier time periods that may be causative.^36,42^ Two sensitivity analyses were conducted to evaluate the final models (see Supplementary Materials and Supplementary Table S1 and Supplementary Table S2). All analyses were adjusted for PMA at MRI scan. Two-sided *P* < 0.05 was considered statistically significant. All analyses were conducted using Stata 17.0 (Stata Corp, College Station, TX).

## Results

Of the original cohort of 395 preterm infants, one infant died at one year of age, and one set of twins were withdrawn from the study by their parents after the term MRI. Of these 392 infants, 31 were diagnosed with PWML, and of these, two were excluded due to motion artifacts and one due to missing T1 images. Thus, 28 patients (7.1%) had high quality images for PWML segmentation and quantification. Table 1 summarizes important antenatal, intrapartum, and postnatal maternal and infant clinical factors for the full cohort prior to term-equivalent age MRI. The mean (SD) birth GA of the sample of infants without and with PWML was 28.8 (2.5) and 29.9 (2.3) weeks, respectively. Intra-rater reliability for our neonatal neuroradiologist for white matter abnormalities (kappa = 0.88) and intra-rater reliability for the PWML segmentations were both excellent (Dice similarity index: 0.85). There was limited correlation between PWML volume and the GBASm score (*r* = −0.034).

We categorized PWML volume (referred to as PWML severity) by thresholding it into terciles. This resulted in categorizing 364 infants with no PWML, 9 with mild, 10 with moderate, and 9 with severe lesions (ordinal; Fig. 1). The categorization of PWML volume was necessary for the antecedent analysis because this data was heavily skewed (93% of subjects had a volume of 0) and there was no data transformation that would allow use to use linear regression analysis without violating the main assumptions of this approach. The choice of terciles over dichotomizing PWML volume data allowed us to preserve more statistical power to identify antecedents. In bivariable analyses controlling for PMA at MRI scan, several antecedent factors were associated with PWML severity at *p* < 0.15, including ANS (p = 0.031), maternal smoking during pregnancy (*p* = 0.145), maternal use of opioids/narcotics during pregnancy (*p* = 0.008), moderate-severe HCA (*p* = 0.072), days on mechanical ventilation (*p* = 0.117), length of parenteral nutrition (*p* = 0.046), and caffeine therapy (*p* = 0.051). Notable non-significant factors included birth weight (*p* = 0.272), GA (*p* = 0.305); sex (*p* = 0.962), 5-minute Apgar score (*p* = 0.757), and intubation at birth (*p* = 0.578).

In multivariable analyses, acute HCA and prenatal narcotics/opioid use increased the odds of developing severe PWML while a complete course of ANS and increasing PMA at MRI scan were associated with decreased odds of developing PWML (Table 2).

**Table 2:** Multivariable ordinal regression model for correlation between antecedent factors and punctate white matter lesion (PWML) severity.

Clinical antecedents	Odds Ratio (95% CI)	*P* Value
Prenatal opioid exposure	4.24 (1.51, 11.88)	*0.006*
Histologic chorioamnionitis (moderate-severe)	2.74 (1.04, 7.20)	*0.041*
Antenatal steroids (complete course)	0.40 (0.18, 0.90)	*0.027*
Postmenstrual age at MRI scan	0.68 (0.51, 0.92)	*0.012*
Birth weight	1.00 (0.99, 1.00)	0.127

Significant values are in italics.

Of the 392 preterm infants, 339 (86%) returned at 2 years CA for neurodevelopmental testing. Of the 336 infants with a complete and valid CP exam, 39 (11.6%) developed CP; 28 (8.3%) were mild; 6 (1.8%) were moderate; and 5 (1.5%) were severe. Of the 28 infants with radiologist confirmed PWML, 6 (21%) developed CP, ranging from mild to severe. The location of PWML for these 6 infants was frontal, parietal, or both; 4 had severe PWML and 2 had mild PWML with one case having co-occurring right sided porencephaly on their MRI. Supplementary Fig. S1 in the Supplementary Materials demonstrates the range of volumes for these 6 infants that developed CP and the remaining 22 that did not with PWML.

In bivariable analyses, increasing severity of PWML was associated with 1.75 odds of developing CP (*p* = 0.012). In multivariable logistic regression analyses PWML severity was significantly predictive of CP (OR 2.12; 95% CI: 1.34, 3.37), independent of several known predictors (Table 3).

**Table 3:** Independent value of punctate white matter lesion (PWML) severity in predicting cerebral palsy at 2 years corrected age in preterm infants.

Predictor	Bivariable Odds Ratio (95% CI)	*P* Value	Multivariable Odds Ratio (95% CI)	*P* Value
PWML severity	1.75 (1.13, 2.69)	0.012	2.12 (1.34, 3.37)	*0.001*
PMA at MRI scan	1.41 (1.08, 1.85)	0.012	1.37 (1.02, 1.85)	*0.039*
Gestational age			0.85 (0.72, 0.999)	*0.049*
Sex (female)			0.52 (0.25, 1.15)	0.111
Moderate-severe BPD			1.20 (0.49, 2.94)	0.694
GBASm			1.14 (1.06, 1.22)	*<0.001*

*PMA* postmenstrual age, *BPD* bronchopulmonary dysplasia, *GBASm* modified global brain abnormality score.

Significant values are in italics.

In secondary bivariable and multivariable linear regression analyses, PWML severity was not independently associated with BSID-III motor, cognitive, or language composite scores at 2 years CA (Table 4). Several other antenatal and NICU variables, most notably, HCA, multiple births, modified GBAS, and high-risk social status were significant at predicting each of the Bayley scores at 2 years CA. Interestingly, PWML volume was a significant predictor of the BSID-III cognitive score (*p* = 0.006) but not motor or language score at 2 years CA (Supplementary Table S3, Supplementary Materials). As in the original analysis, GBASm was the second strongest predictor (after social status) of BSID-III scores with each increase in GBASm score associated with 1-point decrease in BSID-III motor, cognitive, and language scores.

**Table 4:** Punctate white matter lesion (PWML) severity and prediction of Bayley-III Motor, Cognitive, and Language composite scores in multivariable models at 2 years corrected age.

Predictor	Motor Score $\beta$ (95% CI)	*P* Value	Cognitive Score $\beta$ (95% CI)	*P* Value	Language Score $\beta$ (95% CI)	*P* Value
PWML	−1.48 (−3.79, 0.82)	0.206	−1.73 (−4.10, 0.64)	0.151	−1.69 (−4.86, 1.47)	0.293
PMA at MRI scan	−0.74 (−1.77, 0.28)	0.155	−0.77 (−1.82, 0.29)	0.154	−1.12 (−2.53, 0.30)	0.121
Moderate-severe acute HCA	−4.86 (−8.88, −0.85)	*0.018*	−4.30 (−8.41, −0.19)	*0.040*	−7.10 (−12.58, −1.62)	*0.011*
HDP	−1.62 (−4.45, 1.20)	0.259	−2.55 (−5.45, 0.34)	0.084	−2.38 (−6.39, 1.52)	0.231
Antenatal steroids	1.87 (−1.23, 4.97)	0.236	1.65 (−1.53, 4.83)	0.307	2.31 (−1.94, 6.66)	0.285
Multiple birth	−6.05 (−8.97, −3.13)	*<0.001*	−5.06 (−8.05, −2.07)	*0.001*	−6.89 (−10.92, −2.87)	*0.001*
Gestational age	0.52 (−0.13, 1.17)	0.117	0.40 (−0.27, 1.07)	0.236	0.23 (−0.68, 1.13)	0.615
Sex (female)	2.40 (−0.24, 5.04)	0.074	2.00 (−0.69, 4.71)	0.145	5.60 (1.98, 9.23)	*0.003*
Moderate-severe BPD	−1.42 (−5.43, 2.58)	0.485	0.99 (−3.12, 5.96)	0.636	0.81 (−4.70, 6.32)	0.772
Sepsis	−0.75 (−5.32, 3.82)	0.747	−3.38 (−8.05, 1.28)	0.154	−2.80 (−9.02, 3.43)	0.378
GBASm	−1.10 (−1.40, −0.79)	*<0.001*	−1.00 (−1.31, −0.68)	*<0.001*	−1.08 (−1.50, −0.66)	*<0.001*
High-risk social status	−4.92 (−7.63, −2.21)	*<0.001*	−8.43 (−11.21, −5.65)	*<0.001*	−13.40 (−17.15, −9.65)	*<0.001*

*PMA* postmenstrual age, *HCA* histologic chorioamnionitis, *HDP* hypertensive disorders of pregnancy, *BPD* bronchopulmonary dysplasia, *GBASm* modified global brain abnormality score.

Significant values are in italics.

## Discussion

PWML are among the most common types of white matter injuries observed on MRI scans of preterm newborns.^10–16^ In this regional multisite prospective cohort of preterm infants born at $\le$32 weeks GA, using an objective definition of PWML on term-equivalent age MRI, we identified moderate-severe acute HCA and antenatal corticosteroids as novel risk and protective factors, respectively, of PWML. We also found that preterm infants with PWML have 2-fold higher odds per PWML severity category of developing CP, independent of several known predictors. However, PWML were not predictive of motor, cognitive, or language development scores as measured on the BSID-III at 2 years CA. However, in secondary analyses, PWML volume (continuous) did appear to be predictive of BSID-III cognitive scores, independent of other known predictors of neurodevelopment, including the GBASm score.

Although PWML can be seen on MRIs of term infants as incidental findings or with certain conditions,^43^ preterm infants born between 23 to 32 weeks are at highest risk.^24,44^ While a few studies did not find PWML to be predictive of CP,^14^ our findings are consistent with the work of several investigators who reported a significantly increased odds of developing CP in preterm infants with PWML.^17,19,20,22,23^ Unlike most prior studies, we further demonstrated that PWML were independently predictive of CP, over and above other known predictors of CP, including other brain abnormalities on neuroimaging, GA, and sex. Further, increasing severity of PWML from mild to severe was predictive of over 6-fold odds of developing CP. We also objectively measured and quantified PWML severity using manual segmentation that reduced measurement error with resulting improved ability to identify true associations. In secondary analyses, PWML severity was not predictive of Bayley motor, cognitive, or language subtest scores at 2 years CA. Our findings largely agree with prior studies that also did not find a relationship with cognitive or language development but conflict with several studies that reported an association with motor scores at 18–24 months CA in preterm infants.^14,17,19,22^ As with the CP analyses, these studies either did not control for or only controlled for a limited number of predictors. We controlled for 10 known predictors to evaluate the independent predictive value of PWML. Thus, it is possible that PWML do not provide unique information over and above other neuroimaging abnormalities and known clinical predictors of neurodevelopmental outcomes other than CP. Alternatively, the lower prevalence (7.1%) of PWML in our cohort, which was considerably lower than other cohorts, may have limited our statistical power to identify a significant independent relationship with neurodevelopmental outcomes. The lower prevalence may be explained by our later imaging window (39–44 weeks PMA) as compared to prior studies that reported higher rates of PWML since there is a known inverse relationship between PWML and PMA at MRI scan.^13,45^

Our other primary objective was to examine antenatal factors associated with PWML. We identified two risk factors and one protective factor as being independently associated with the development of objectively quantified PWML severity. One of these risk factors—moderate-severe acute HCA—has not been previously associated with the risk of developing PWML, and antenatal corticosteroids, the protective factor, has not been previously identified as being associated with reduced risk of PWML. No prior studies have reported HCA as an independent risk factor for PWML. However, many studies control for GA in the model as a confounder rather than treating it as a mediator of the relationship between HCA and brain injury or NDI.^46,47^ This results in collider stratification bias and can negate a potential significant association.^35,48^ While human data is lacking, a rabbit model of intrauterine infection demonstrated focal rarefaction and disorganization of white matter that resembled human neonatal brains with focal necrosis.^49^ Using this same cohort, we previously reported a significant negative relationship between HCA and a composite measure of brain abnormalities at term that was inclusive of PWML.^35^ From the same cohort, we also demonstrated a significant adverse association between prenatal opioid exposure and PWML, independent of other antenatal exposures/confounders.^30^ This builds on previous literature that has found associations between prenatal opioid exposure and PWML in term infants^26,50,51^ and contrasts with other work that has not observed white matter injuries in infants with opioid exposure.^52,53^ Previous work has linked prenatal opioid use and prenatal tobacco exposure to CP and other neurodevelopmental outcomes.^54,55^ Studies that used mouse models^56,57^ to examine prenatal exposure to methadone, have shown altered cortical and subcortical regions, altered brain microstructure, and decreased cerebral neurometabolite levels. These effects on neurite morphology may explain the adverse neurodevelopmental effects seen in children with prenatal opioid exposure.^58,59^ We also identified an inverse relationship between antenatal corticosteroid therapy (complete course) and PWML. In line with our findings, one study reported a significant association between antenatal corticosteroids and PWML in preterm infants,^60^ but another did not.^15^ Meta-analyses of randomized trials of antenatal corticosteroids in extremely preterm infants have demonstrated reduced risk of brain injuries (e.g., periventricular leukomalacia; severe intraventricular hemorrhage) and NDI, suggesting the association we identified is valid.^61^ A recent large study identified a protective association between antenatal magnesium as well as postnatal prophylactic indomethacin (short but not long duration) exposure and odds of PWML over time between two sequential cohorts.^62^ In our cohort, we did not find an association between these two clinical factors and PWML, possibly due to differences in study design and population (e.g., cross-sectional analysis, single model that prioritized earlier antenatal factors over later postnatal factors, and a more representative cohort of infants from all regional academic and non-academic institutions).

There are limitations of this study. Although our cohort included 392 infants, only 28 had PWML on MRI at term. Nevertheless, most of our findings externally validate the observations of prior cohort studies. Prenatal opioid use was self-reported by mothers instead of being biochemically validated; this may have led to under-reporting. However, this indicates that if the prevalence of exposure was higher, then the effects of opioids may have been even more strongly associated with PWML. Further, we did not examine the effects of postnatal opioid exposure during the NICU stay on PWML. This important question remains unexplored and deserves additional study. Use of stepwise selection to identify predictors generally has the problem of optimizing variables to fit a specific dataset but can have limited generalizability. Therefore, independent validation of HCA, the main novel antecedent of PWML we identified, is required. Last, Bayley outcomes at 2 years CA are poor predictors of pediatric developmental outcomes,^52,53,63,64^ thus, these secondary analyses require additional study.

Our study also had several strengths. Unlike most prior studies, we quantified PWML volume reliably, thus resulting in reduced measurement error and increased study power. We also created multivariable regression models in which we ordered over 50 clinical risk and protective factors temporally, so that the earliest occurring factors were entered first and could not be displaced by later occurring covariates. Prior antecedent studies examined only univariate relationships between exposure and PWML or only controlled for a few clinical confounders. Similarly, each of our prognostic models included up to 10 known predictors to robustly identify the independent ability of PWML to predict neurodevelopmental outcomes at 2 years CA.

In conclusion, we found that increasing severity of PWML when quantified using conventional brain MRI at term, is associated with modifiable antenatal risk factors and an increased risk of CP diagnosis in infants born $\le$32 weeks’ GA. Identifying additional modifiable risk factors of PWML would help promote the prevention of motor abnormalities and increase quality of life for infants born very preterm. Further validation of our findings with longer-term follow-up is warranted.

## Supplementary information

Supplementary Materials
