# Supplementary material for: Severity of punctate white matter lesions in preterm infants: antecedents and cerebral palsy prediction
Source: Pediatr Res. 2025 May 30;98(6):2220–7. doi: 10.1038/s41390-025-04157-z (PMC12811139; doi:10.1038/s41390-025-04157-z)
Supplement: Supplementary file 1 — Supplementary Materials [file 41390_2025_4157_MOESM1_ESM.pdf]

Supplementary Materials

Severity of Punctate White Matter Lesions in Preterm Infants: Antecedents and Cerebral Palsy Prediction

E. Melinda Mahabee-Gittens[1,2]; Venkata Sita Priyanka Illapani[3,4]; Beth M. Kline-Fath[2,3,5]; Karen Harpster[3,6,7]; Ashley Magnino[1]; Stephanie Merhar[2,3,4]; Nehal A. Parikh[2,3,4]; for the Cincinnati Infant Neurodevelopment Early Prediction Study (CINEPS) Investigators

Supplemental Methods

For multivariable logistic regression models, the number of predictors to outcomes is widely regarded as optimal between 5 to 10 cases of the outcome per independent variable (predictor).[1,2] This recommendation aims to ensure sufficient statistical power, model stability, and reliable estimation of regression coefficients, while avoiding overfitting. We performed a sensitivity analysis to ensure that our selection of six predictors did not result in unstable estimates and/or overfitting. We did this by removing the two variables – sex and moderate-severe bronchopulmonary dysplasia – from the full model that were not significant (Supplemental Table S1).

We performed a second sensitivity analysis to evaluate if three key antenatal predictors of CP – moderate-severe histologic chorioamnionitis, prenatal opioid use, and receiving antenatal corticosteroid – would displace punctate white matter lesion (PWML) as an independent predictor of cerebral palsy at 2 years corrected age in preterm infants. To minimize changes of overfitting as in the analysis above, we removed the two postnatal predictors there were not significant in the full CP model (moderate-severe bronchopulmonary dysplasia and sex) (Supplemental Table S2). To further ensure that we were not overfitting our model, we tested each variable one by one (vs. all three at the same time) in the model with the four postnatal predictors.

Supplemental Results

In a sensitivity analysis that removed two predictors that were non-significant in the full CP model (thus changing the ratio of cases to predictors from 6.5 to 10), the ORs and 95% CIs for the four predictors remained nearly identical (right column) as compared to the full model (left) (Supplemental Table S1).

In the second sensitivity analysis, inclusion of antenatal predictors of CP did not impact the independent ability of PWML severity to predict CP at age 2 (Supplemental Table S2). The OR and 95% CI for PWML severity and P values for the three antenatal predictors remained nearly the same in models that tested each variable one by one (results not shown).

Supplemental Table S1. Sensitivity analysis where number of predictors was limited to four (right column) rather than six (left column; full model) to assess the independent value of punctate white matter lesion (PWML) severity in predicting cerebral palsy at 2 years corrected age in preterm infants.

**Predictor**	**Multivariable Odds Ratio (95% CI): full model**	***P* Value**	**Multivariable Odds Ratio (95% CI)**	***P* Value**
PWML severity	2.12 (1.34, 3.37)	*.001*	2.09 (1.32, 3.33)	*.002*
PMA at MRI scan	1.37 (1.02, 1.85)	*.039*	1.36 (1.02, 1.83)	*.036*
Gestational age	0.85 (0.72, 0.999)	.049	0.84 (0.72, 0.98)	.026
GBASm	1.14 (1.06, 1.22)	<.001	1.14 (1.07, 1.22)	<.001
Moderate-severe BPD	1.20 (0.49, 2.94)	.694		
Sex (female)	0.52 (0.25, 1.15)	.111		

Abbreviations: PMA – postmenstrual age; GBASm – modified global brain abnormality score

Supplemental Table S2. Sensitivity analysis to evaluate if antenatal predictors of CP would displace punctate white matter lesion (PWML) as an independent predictor of cerebral palsy at 2 years corrected age in preterm infants.

**Predictor**	**Multivariable Odds Ratio (95% CI): full model**	***P* Value**	**Multivariable Odds Ratio (95% CI)**	***P* Value**
PWML severity	2.12 (1.34, 3.37)	*.001*	2.05 (1.24, 3.39)	*.005*
PMA at MRI scan	1.37 (1.02, 1.85)	*.039*	1.37 (1.03, 1.83)	*.031*
Gestational age	0.85 (0.72, 0.999)	.049	0.84 (0.72, 0.98)	.026
GBASm	1.14 (1.06, 1.22)	<.001	1.14 (1.07, 1.23)	<.001
Moderate-severe BPD	1.20 (0.49, 2.94)	.694		
Sex (female)	0.52 (0.25, 1.15)	.111		
Prenatal opioid exposure			1.76 (0.60, 5.22)	.306
HCA (moderate-severe)			0.58 (0.21, 1.60)	.289
ANS (complete course)			0.76 (0.33, 1.73)	.512

Abbreviations: PMA – postmenstrual age; GBASm – modified global brain abnormality score; HCA – histologic chorioamnionitis; ANS – antenatal corticosteroids

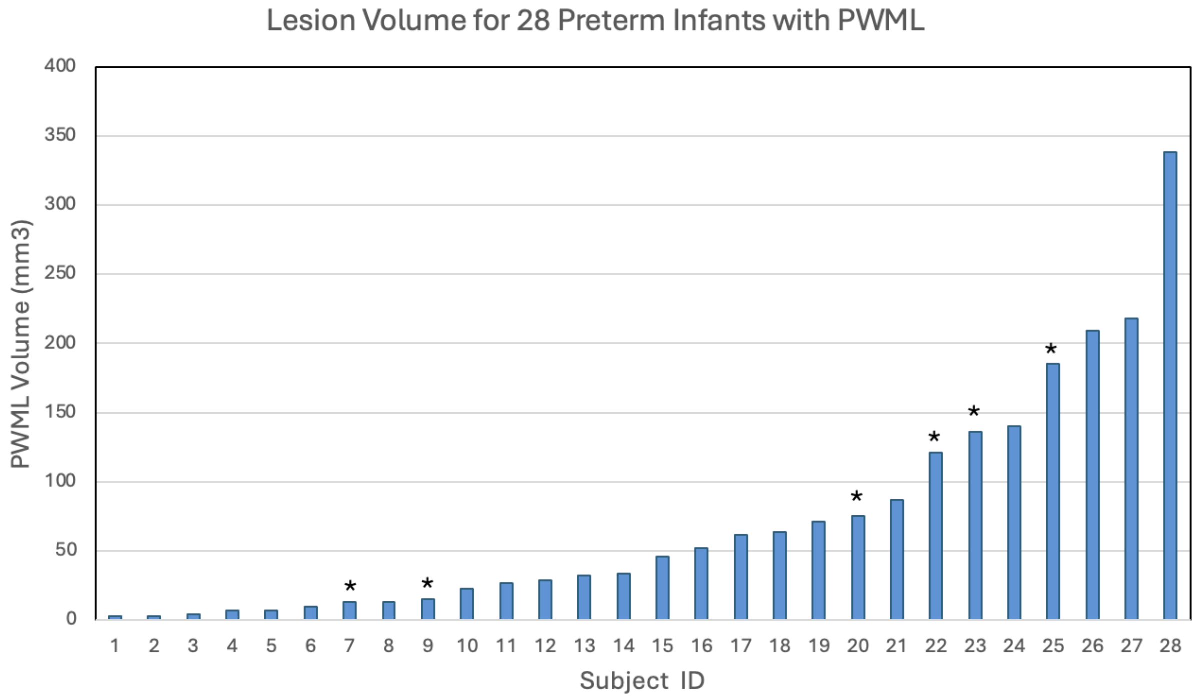

Supplemental Figure S1. Lesion volume for 28 preterm infants diagnosed with PWML on MRI at term-equivalent age and CP follow-up testing. Lesions were segmented manually and was calculated as the product of voxel volume and total number of voxels in the detected PWML. Asterisks (*) represent the six infants from the cohort that developed CP at 2 years corrected age.

Supplemental Table S3. Punctate white matter lesion (PWML) volume and prediction of Bayley-III Motor, Cognitive, and Language composite scores in multivariable models at 2 years corrected age.

Predictor	Motor Score β (95% CI)	*P* Value	Cognitive Score β (95% CI)	*P* Value	Language Score β (95% CI)	*P* Value
PWML volume (mm^3)	-0.05 (-0.12, 0.01)	.105	-0.06 (-0.11, -0.02)	*.006*	-0.07 (-0.15, 0.01)	.081
PMA at MRI scan	-0.72 (-1.79, 0.35)	.189	-0.73 (-1.76, 0.29)	.159	-1.09 (-2.50, 0.33)	.133
Moderate-severe acute HCA	-4.74 (-8.80, -0.69)	*.022*	-4.15 (-8.29, -0.01)	*.049*	-6.90 (-11.99, -1.82)	*.008*
HDP	-1.48 (-4.38, 1.43)	.318	-2.38 (-5.20, 0.43)	.097	-2.17 (-5.93, 1.58)	.256
Antenatal steroids	1.83 (-1.39, 5.05)	.265	1.60 (-1.55, 4.74)	.317	2.23 (-1.96, 6.41)	.296
Multiple birth	-6.10 (-9.06, -3.14)	*<.001*	-5.12 (-8.00, -2.23)	*.001*	-6.95 (-11.00, -2.90)	*.001*
Gestational age	0.54 (-0.09, 1.17)	.094	0.42 (-0.28, 1.13)	.239	0.25 (-0.60, 1.11)	.561
Sex (female)	2.39 (-0.18, 4.95)	.068	1.99 (-0.67, 4.65)	.142	5.57 (1.96, 9.18)	*.003*
Moderate-severe BPD	-1.51 (-5.51, 2.49)	.459	0.89 (-3.26, 5.03)	.675	0.69 (-4.62, 6.01)	.798
Sepsis	-0.79 (-4.87, 3.28)	.702	-3.44 (-7.80, 0.92)	.122	-2.87 (-8.26, 2.51)	.295
GBASm	-1.11 (-1.42, -0.79)	*<.001*	-1.01 (-1.29, -0.73)	*<.001*	-1.09 (-1.46, -0.72)	*<0.001*
High-risk social status	-4.78 (-7.43, -2.13)	*<.001*	-8.27 (-10.86, -5.67)	*<.001*	-13.21 (-16.79, -9.63)	*<0.001*

Abbreviations: PMA – postmenstrual age; HCA – histologic chorioamnionitis; HDP – hypertensive disorders of pregnancy; BPD – bronchopulmonary dysplasia; GBASm – modified global brain abnormality score

References

1. Peduzzi P, Concato J, Kemper E, Holford TR, Feinstein AR. A simulation study of the number of events per variable in logistic regression analysis. *Journal of clinical epidemiology*. Dec 1996;49(12):1373-9. doi:10.1016/s0895-4356(96)00236-3
2. Vittinghoff E, McCulloch CE. Relaxing the rule of ten events per variable in logistic and Cox regression. *American journal of epidemiology*. Mar 15 2007;165(6):710-8. doi:10.1093/aje/kwk052
